# Brucellosis Presenting With Pancytopenia and Foot Drop

**DOI:** 10.7759/cureus.10293

**Published:** 2020-09-07

**Authors:** Asad Inayat, Qudrat U Marwat, Waqar Hayat, Muhammad S Faisal

**Affiliations:** 1 Internal Medicine, Khyber Teaching Hospital, Peshawar, PAK; 2 Internal Medicine, Portiuncula University Hospital, Galway, IRL; 3 Medicine, Fauji Foundation Hospital, Peshawar, PAK; 4 Pharmacology, Khyber Medical College, Peshawar, PAK

**Keywords:** neurobrucellosis, foot drop, brucellosis, pancytopenia

## Abstract

Brucellosis is a zoonotic bacterial infection and is highly treatable. Despite being eradicated from most developed countries, its prevalence in Africa, the Middle East and parts of Asia pose a high risk of morbidity if undiagnosed. It mainly affects the reticuloendothelial system and manifests as fever, constitutional symptoms, hepatomegaly, and splenomegaly, but other organ systems can also be involved. Neurobrucellosis is a type of brucellosis in which the central or peripheral nervous systems are affected. It can present with a myriad of signs and symptoms such as stroke, meningitis, and cranial nerve palsies. We present a rare case of a 16-year-old boy with brucellosis who presented with pancytopenia and foot drop. He was subsequently managed with intravenous antibiotics for brucellosis and physiotherapy for foot drop.

## Introduction

Brucellosis is a very important zoonotic infections. Although it has been eradicated from the developed world, this disease is still prevalent in Africa, the Mediterranean region, the Middle East, and parts of Asia and Latin America [1]. It is caused by the bacterial genus Brucella which is a Gram-negative bacteria. It is mainly transmitted from animals to humans by the ingestion of contaminated food, direct contact with infected animals, or inhalation of infected particles. [2].

The most common presentation of brucellosis is fever. It can present as acute, subacute, and chronic. Anorexia, fatigue, malaise, body aches, sweating, and arthralgia are also common symptoms. Brucella mainly affects the reticuloendothelial system and can result in hepatosplenomegaly and lymphadenopathy, which are common physical examination findings in these patients. It can spread to any organ system, but infection of the bones and joints is most common [3]. Neurobrucellosis is the term used when the infection spreads to the nervous system. Neurobrucellosis can present with stroke, idiopathic intracranial hypertension, meningitis, encephalitis, and cranial nerve palsies. Foot drop is the cranial nerve palsy discussed in this case report. Prompt treatment with antibiotics is crucial to avoid irreversible damage.

## Case presentation

A 16-year-old boy with no significant past medical history presented to the emergency department (ED) of the Khyber teaching hospital in Peshawar in shock. At presentation, his blood pressure was 70/50 mmHg, his heart rate was 120 beats per minute, his temperature was 101°F, and his Glasgow Coma Scale score was 15/15. His initial laboratory workup showed features of pancytopenia with hemoglobin of 4 g/dL (reference range: 13.5-17.5 g/dL). He was immediately resuscitated with intravenous (IV) fluids and blood transfusions in the ED. His condition stabilized after receiving three units of blood transfusion and 2L normal saline. He was shifted to the internal medicine ward for workup of his pancytopenia.

The detailed physical examination in the internal medicine ward revealed pallor and fever (body temperature: 101°F). The findings of his cardiac and pulmonary examinations were unremarkable. Evidence of hepatosplenomegaly was noted on his abdominal exam, and his neurological exam revealed difficulty with dorsiflexion of the right foot (Power: 0/5). He had a steppage gait favoring foot drop.

The laboratory investigation showed evidence of pancytopenia. His hemoglobin 7 g/Dl (reference range: 13.5-17.5 g/Dl), his white blood cell count was 3,000/ml (reference range: 4000-11,000/ml), and his platelet count was 75,000/ml (reference range: 150,000- 400,000/ml). His malaria parasite smear and Dengue serology investigation were negative (these investigations were done first because they are the more common causes of pancytopenia in Pakistan). His reticulocyte count was healthy, and there was no evidence of hemolysis. The results of his liver function test, urea, creatinine, and chest x-ray were all unremarkable. Three sets of blood cultures from different sites were collected and sent to the laboratory before starting the patient on empirical antibiotics. The results of his electrocardiogram and echocardiography were also within reference ranges. His ultrasound showed hepatomegaly (12 cm) and splenomegaly (15 cm). His nerve conduction study (NCS) showed decreased amplitude and prolonged latency. His electromyography (EMG) showed features of denervation. He was started on IV ceftriaxone 1 g twice daily on the first day.

A detailed history revealed that his father is a farmer, and they have domesticated animals like cows and goats at home. Based on this history, Brucella serology titers were sent, revealing >1:160 (less than 1:80 is negative) suggestive of brucellosis. He was started on oral doxycycline 100 mg twice daily, and streptomycin 500 mg via intramuscular (IM) injection once daily were added to ceftriaxone on his second day of care. His blood culture demonstrated the growth of Brucella melitensis on day 14. He received ceftriaxone, doxycycline, and streptomycin for four weeks.

After four weeks, his signs and symptoms showed marked improvement with no evidence of pancytopenia. His hemoglobin had increased to 12 g/dl, his total leukocyte count was 6,000/ml, and his platelet count was 210,000/ml. Moreover, there was no evidence of hepatosplenomegaly on ultrasound examination. He remained afebrile for the last two weeks. Daily physiotherapy and exercises were arranged to address his foot drop, which was the only issue yet remaining. Ceftriaxone and streptomycin were stopped after four weeks, and the patient was sent home on doxycycline 100 mg twice daily and rifampicin 600 mg once daily. He was advised to follow-up for an outpatient visit and physiotherapy. After receiving this therapy for further eight weeks, his right leg function showed an 80% increase in function (Power: 4/5), and there was no evidence of foot drop on examination. His antibiotics were stopped, and only physiotherapy was advised for further management.

## Discussion

The diagnosis of brucellosis requires prompt history taking. Dietary, occupational, and travel history are very important for ordering the investigation for this disease. Drinking unpasteurized milk is the most important source of transmission from cattle to humans. Brucella commonly affects the reticuloendothelial system, so pancytopenia is a frequent presentation in both adults and children [4]. The definitive diagnosis of brucellosis is based on clinical findings, serological testing, and cultures from the blood and bone marrow. Sensitivity of bone marrow culture is 80% to 90% [2]. In patients with neurobrucellosis, cerebrospinal fluid (CSF) shows lymphocytic pleocytosis in 88% to 99% of cases, but culture is positive in 50% of cases [2].

Prompt antibiotics treatment is very crucial for treating the active disease and preventing complications and relapse. Multidrug regimen antibiotics are preferred as compared to monotherapy because of the risk of relapse. Doxycycline, rifampicin, streptomycin, gentamicin, and trimethoprim-sulfamethoxazole are the common antibiotics used for the treatment of brucellosis [5].

Brucellosis with neurological involvement occurs in a small number of patients. According to one study, neurological consequences occur in 6.1% of cases of brucellosis [6]. Common presentations of neurobrucellosis are neck rigidity (46%), radiculopathies of the legs(42%), cranial nerve involvement (25%), disorientation (19.5%), and behavioral disorders (18.3%) [6]. The most common cranial nerve palsies involve cranial nerves seven and eight [6].

Foot drop is a very rare presentation of neurobrucellosis, and according to one study in Turkey, foot drop occurs in 0.09% cases [7]. In addition to the neurological involvement, the typical findings in our patient in favor of brucellosis were fever, constitutional symptoms, hepatosplenomegaly, and pancytopenia.

The diagnosis of neurobrucellosis was challenging in our case as both the CSF brucella titers and culture were negative. Based on the clinical presentation and positive serological titers of >1:160 and positive blood culture, we are confident our patient had a complication of brucellosis with peroneal nerve involvement resulting in foot drop. According to one study, CSF brucella titer of >1:8 showed a sensitivity of 94% and specificity of 96%. Therefore, if the titers are elevated, even in small numbers, the diagnosis is very likely neurobrucellosis. CSF cultures were positive only in 15% of cases [8]. Lymphocytic pleocytosis can be found in patients with neurobrucellosis.

The standard therapy for brucellosis is oral doxycycline combined with intramuscular streptomycin [9-12]. The other antibiotics used for brucellosis are rifampicin and trimethoprim-sulfamethoxazole. Ceftriaxone has also been used successfully against Brucella as it is a Gram-negative bacteria; this was the reason that we initially used it along with doxycycline and streptomycin [10]. Timely treatment with antibiotics is essential to prevent complications and relapses. In our case, we used IV ceftriaxone 1 g twice daily, oral doxycycline 100 mg twice daily, and IM streptomycin 500 mg once daily for four weeks, followed by oral doxycycline 100 mg once daily, and oral rifampicin 600 mg once daily for another eight weeks, which resulted in the resolution of both pancytopenia and foot drop. Physiotherapy and exercises were also important for the treatment of foot drop.

The presence of pancytopenia and foot drop is very rare finding; it can be two separate entities, but the NCS, EMG, and radiological testing found no other possible cause for foot drop. Based on the patient’s history, investigation, and response to treatment, we can say that this case represents a complication of brucellosis.

## Conclusions

Brucellosis most commonly affects the reticuloendothelial system, but it can spread to any organ in the body. A prompt diagnosis of brucellosis is necessary and it should be considered as a differential diagnosis in fever of unknown origin. Importantly, neurobrucellosis is a very rare but lethal complication of brucellosis, and it can cause a number of complications ranging from cranial nerve palsies to serious ones like meningitis and encephalitis. The early recognition and management of neurobrucellosis (foot drop) with a multidrug regimen and effective physiotherapy is critical to prevent permanent damage.
